# Foot Arch Status and Muscular Strength: Associations with Unilateral Balance and Physical Performance

**DOI:** 10.3390/diagnostics16142279

**Published:** 2026-07-21

**Authors:** Mansour Abdullah Alshehri, Mohammed S. Alghamdi, Dimah Ayed Alsaedi, Aseel Talal Alsharif, Najat Essam Alotaibi, Hammad Alhasan, Ammar Fadil, Moayad S. Subahi, Moiyad S. Aljehani, Abdulaziz Awali, Mohamed Salaheldien Mohamed Alayat, Moaz Tobaigy

**Affiliations:** 1 Department of Medical Rehabilitation Sciences, Faculty of Applied Medical Sciences, Umm Al-Qura University, Makkah 24382, Saudi Arabiahshasan@uqu.edu.sa (H.A.); asfadil@uqu.edu.sa (A.F.); mssubahi@uqu.edu.sa (M.S.S.); msjehani@uqu.edu.sa (M.S.A.); amawali@uqu.edu.sa (A.A.); msayiat@uqu.edu.sa (M.S.M.A.); 2 Prosthetics and Orthotics Department, Faculty of Medical Rehabilitation Sciences, King Abdulaziz University, Jeddah 21589, Saudi Arabia; mtobaigy@kau.edu.sa

**Keywords:** foot posture, medial longitudinal arch, navicular drop test, handgrip strength, single-leg stance test, balance, postural control, 10 m walk test, walking speed, 30 s chair stand test

## Abstract

**Background/Objectives**: Foot arch status and muscular strength may influence balance and physical performance. However, evidence regarding their associations with unilateral balance performance, walking performance, and lower limb functional capacity in healthy young adults, particularly in Saudi Arabia, remains limited. This study investigated the associations of foot arch status and handgrip strength with these outcomes in healthy young Saudi adults. **Methods**: In this cross-sectional study, 104 healthy young adults (mean age: 21.1 ± 1.2 years; 52.9% female) underwent assessment of foot arch status using the sit-to-stand navicular drop test (SSNDT) and of muscular strength using a digital hand dynamometer. Unilateral balance performance was assessed using the single-leg stance test (SLST) under eyes open and eyes closed conditions. Walking performance and lower limb functional capacity were assessed using the 10 m walk test (10-MWT) and the 30 s chair stand test (30 s-CST), respectively. Generalised estimating equations and multivariable linear regression models were used to examine adjusted associations. **Results**: Greater SSNDT scores were independently associated with shorter SLST duration for both the right (β = −0.46, *p* = 0.003) and left feet (β = −0.25, *p* = 0.048). Greater handgrip strength was independently associated with longer SLST duration for the right foot only (β = 0.17, *p* = 0.019). Neither SSNDT score nor handgrip strength was independently associated with 10-MWT or 30 s-CST outcomes after adjustment. Athletic participants demonstrated superior balance and chair-stand performance, while the male sex was associated with longer SLST duration. **Conclusions**: Greater functional lowering of the medial longitudinal arch was independently associated with poorer unilateral balance performance, whereas handgrip strength demonstrated a more limited association. These findings suggest that foot arch behaviour is more strongly related to balance performance than to walking performance or lower limb functional capacity.

## 1. Introduction

Foot posture and muscular strength are important determinants of movement efficiency, postural balance control, and musculoskeletal function. The medial longitudinal arch (MLA) plays a key role in load distribution, shock absorption, and force transmission during standing and locomotion [1,2,3]. Variations in MLA structure and function may alter lower limb biomechanics and influence functional performance outcomes [4,5,6], particularly tasks requiring postural stability and neuromuscular control. Recent evidence further supports the importance of foot structure in movement biomechanics, demonstrating that structural foot deformities and foot posture are associated with altered lower limb loading patterns and increased tissue-specific biomechanical loading [7].

Foot posture is commonly classified as pronated, neutral, or supinated according to MLA characteristics [4,8]. Pronated foot posture, characterised by greater functional lowering of the MLA, has been associated with altered plantar pressure distribution [5], modified lower limb biomechanics [9,10], altered neuromuscular responses [11,12], impaired postural balance control [8], and changes in gait characteristics [13]. These biomechanical alterations may influence balance and physical performance through changes in sensory input, neuromuscular control, and lower limb alignment.

Among the available clinical measures, the sit-to-stand navicular drop test (SSNDT) [14] is a modified version of the traditional navicular drop test (NDT) [15]. The SSNDT quantifies functional lowering of the MLA during weight bearing activities [14] and has demonstrated greater measurement consistency and reliability than the traditional NDT [14,16], making it a robust clinical measure of functional foot arch behaviour. Previous evidence suggests that non-neutral foot posture is associated with several musculoskeletal disorders and lower limb injuries [17,18,19,20,21,22,23,24,25,26]. These findings suggest that alterations in foot posture may also influence postural balance and physical performance.

In addition to foot posture, muscular strength may also influence postural balance and physical performance. Handgrip strength is widely recognised as a practical and reliable surrogate measure of overall muscular strength [27,28,29]. It has demonstrated moderate-to-strong correlations with lower limb muscle strength in both young healthy adults [30] and community-dwelling older adults [31]. Furthermore, greater handgrip strength has been associated with better postural control in community-dwelling older adults [31]. As an indicator of general muscular capacity, handgrip strength may therefore provide insight into balance and physical performance.

Postural balance is essential for many everyday activities requiring single limb support, such as walking, stair negotiation, and directional changes [32,33]. The single-leg stance test (SLST) is a widely used measure of unilateral balance performance [32,33,34]. Because unilateral stance imposes greater postural demands than bilateral stance, the SLST may be more sensitive to subtle impairments in balance and lower limb function [34]. Successful SLST performance requires the integration of visual, vestibular, and proprioceptive information [35], with coordinated neuromuscular control of the trunk and lower limbs [36,37,38]. In addition to balance performance, walking speed [39] and lower limb functional capacity [40] are recognised indicators of physical function and can be assessed using the 10 m walk test (10-MWT) [41] and 30 s chair stand test (30 s-CST) [40,42].

Despite growing evidence linking foot posture and muscular strength to balance performance and physical function, several important gaps remain. Many previous studies have been conducted in highly selected populations, including athletes, individuals within restricted body mass index (BMI) ranges, or single-sex cohorts, which may limit the generalisability of their findings. In addition, limited statistical adjustment for important confounding factors, including age, sex, BMI, athletic status, and limb dominance, may have influenced previous findings. Furthermore, many balance studies have assessed performance only under eyes open conditions, limiting understanding of the influence of foot posture under different sensory demands.

These knowledge gaps are particularly relevant in Saudi Arabia, where musculoskeletal disorders represent a substantial health burden. Previous Saudi studies have reported associations between foot posture and low back pain [43] and knee osteoarthritis [44]. However, no Saudi study has examined the associations between foot arch behaviour assessed using the SSNDT and unilateral balance performance under both eyes open and eyes closed conditions. In addition, the relationships between handgrip strength and unilateral balance, walking performance, and lower limb functional capacity remain poorly understood in healthy Saudi adults.

Therefore, the primary aim of this study was to investigate the associations between foot arch status, assessed using the SSNDT, and unilateral balance performance, assessed using the SLST under eyes open and eyes closed conditions, in healthy young Saudi adults. Secondary aims were to examine the associations of foot arch status with walking performance and lower limb functional capacity, assessed using the 10-MWT and 30 s-CST, respectively. In addition, the associations between handgrip strength and all balance and physical performance outcomes were examined.

## 2. Materials and Methods

### 2.1. Study Design and Ethics

This cross-sectional observational study investigated the associations of foot arch status and muscular strength with balance and physical performance outcomes among healthy young adults in Saudi Arabia. This study was conducted and reported in accordance with the Strengthening the Reporting of Observational Studies in Epidemiology (STROBE) guidelines. Participants were recruited using convenience sampling through electronic advertisements, recruitment posters, and direct outreach within the Umm Al-Qura University. All assessments were conducted in the Biomechanics Laboratory, Department of Medical Rehabilitation Sciences, Faculty of Applied Medical Sciences, Umm Al-Qura University, Makkah, Saudi Arabia. The study was approved by the Institutional Review Board of Umm Al-Qura University (ID: HAPO-02-K-012-2025-12-3052) and conducted in accordance with the Declaration of Helsinki. Written informed consent was obtained from all participants prior to participation.

### 2.2. Eligibility Criteria

Eligible participants were healthy adults aged 18 years or older who were able to stand and ambulate independently without substantial physical assistance. Both males and females were eligible for inclusion. Participants were required to understand and follow verbal instructions and to provide written informed consent prior to participation. Participants were excluded if they had any condition that could compromise participant safety or interfere with the accurate assessment of the study exposures or outcomes. Exclusion criteria included lower limb injury, fracture, or surgery within the preceding six months; vestibular disorders; severe musculoskeletal deformities affecting foot posture, mobility, or balance; neurological, cardiovascular, or respiratory conditions that could affect balance or physical performance; pregnancy; and active foot wounds or ulcers that prevented barefoot assessment.

### 2.3. Assessment Standardisation

All assessments were completed during a single laboratory session lasting approximately 45–60 min. Data collection was conducted by trained physiotherapists who had received standardised training in all assessment procedures and demonstrated competency in the required measurement techniques before study commencement. To enhance measurement consistency and minimise inter-assessor variability, assessor roles were standardised throughout the study. One assessor collected demographic and health-related information, a second assessor performed all exposure-related measurements, and two assessors were responsible for outcome assessments, with one recording data and the other providing participant instructions, procedural guidance, and safety monitoring. All exposure and outcome assessments were performed barefoot to standardise testing conditions and minimise the potential influence of footwear on foot posture [14] and balance performance [32,45].

### 2.4. Exposure and Outcome Variables

All exposure and outcome assessments were performed during a single testing session using a standardised assessment sequence for all participants. The assessment included demographic and anthropometric assessments, followed by the SLST, handgrip strength, 10-MWT, and 30 s-CST.

#### 2.4.1. Demographic and Health-Related Variables

Demographic, health-related, and anthropometric data were collected prior to the exposure and outcome assessments. Recorded demographic characteristics included age, sex, nationality, marital status, place of residence, educational status, athletic status (i.e., regular participation in sports or structured physical training), dominant hand, and dominant lower limb. Participants also reported relevant medical conditions and comorbidities to verify study eligibility and confirm compliance with the predefined inclusion and exclusion criteria.

Anthropometric assessments included standing height, body weight, and BMI. Standing height was measured in centimetres using a wall-mounted stadiometer, and body weight was measured in kilograms using a calibrated digital weighing scale. BMI was subsequently calculated as body weight in kilograms divided by height in metres squared (kg/m^2^).

#### 2.4.2. Foot Arch Status

Foot arch status was the primary exposure variable and was assessed using the SSNDT, a clinical measure of functional lowering of the MLA during weight bearing [14]. Previous studies have demonstrated moderate-to-good reliability of the SSNDT, although reliability estimates vary according to the study population and testing protocol, with reported intra-rater intraclass correlation coefficients (ICCs) ranging from 0.68 to 0.70, an inter-rater ICC of 0.72 in healthy adults [14], and a within-day ICC of 0.82 in physically active healthy adults [16].

**Figure 1:**
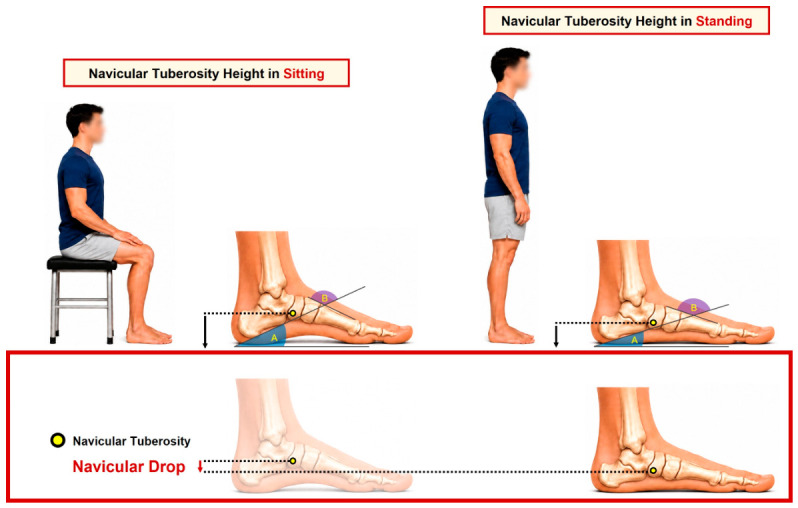
Sit-to-stand navicular drop test (SSNDT) procedure and radiographic indicators of sagittal plane foot alignment. Navicular drop was calculated as the difference between navicular height measured in sitting and relaxed bilateral standing positions. Higher SSNDT scores indicate greater functional lowering of the medial longitudinal arch (MLA) during weight bearing and a more pronated foot posture. Radiographic indicators include the calcaneal inclination angle (A) and calcaneal-first metatarsal angle (B), where lower calcaneal inclination angles and greater calcaneal-first metatarsal angles indicate reduced MLA height and increased foot pronation.

Participants were initially positioned sitting with the hips and knees flexed to approximately 90° and the ankles maintained in a neutral position (0° ankle dorsiflexion). The most prominent aspect of the navicular tuberosity was identified and marked. Navicular height was then measured as the vertical distance between the navicular tuberosity and the supporting surface using a ruler positioned perpendicular to the floor. Participants subsequently assumed a relaxed bilateral standing posture with equal weight distribution across both lower limbs, and navicular height was measured again using the same procedure. The SSNDT score was calculated as the difference between seated and standing navicular height measurements and expressed in millimetres. Higher SSNDT scores indicated greater functional lowering of the MLA during weight bearing and, therefore, a more pronated foot posture [14,15]. Measurements were performed separately for the right and left feet. An illustration of the SSNDT procedure and associated radiographic indicators of foot posture is presented in Figure 1.

#### 2.4.3. Handgrip Strength

**Figure 2:**
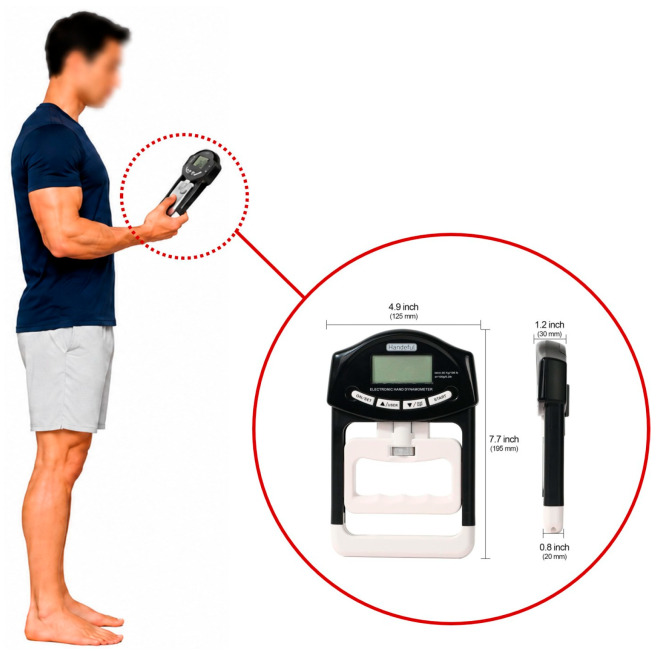
Participant positioning and upper limb alignment during handgrip strength assessment using a digital hand dynamometer.

Handgrip strength was assessed as a secondary exposure variable using a digital hand dynamometer (HFEH11BK; Handeful, Hongkong, China; Figure 2) and was used as a surrogate measure of overall muscular strength. Although the Jamar dynamometer is widely regarded as the reference standard for handgrip strength assessment [46,47,48] previous studies have demonstrated excellent test–retest reliability (ICC = 0.94–0.95) [49] and good concurrent validity relative to the Jamar dynamometer (ICC = 0.82–0.83) [50] for commercially available digital hand dynamometers.

Participants were assessed standing with the shoulder adducted, elbow flexed to 90°, forearm supinated, and wrist maintained in a neutral position [47]. Prior to formal testing, participants received standardised instructions and completed one familiarisation trial. Participants were instructed to avoid compensatory arm or trunk movements during testing. Handgrip strength was then assessed by performing a maximal 5 s isometric contraction using each hand separately. A 60 s rest interval was provided between assessments to minimise fatigue. Maximum handgrip strength was recorded in kilograms for each hand separately. Participant positioning and upper limb alignment during handgrip strength assessment are illustrated in Figure 2.

#### 2.4.4. Unilateral Balance Performance

Unilateral balance performance was assessed as the primary outcome variable using the SLST under both eyes open and eyes closed conditions. Both visual conditions were included because visual input is an important contributor to postural balance control, and SLST performance has been shown to vary according to visual condition and sensory availability [32,51]. The testing protocol was based on previously published normative SLST procedures implemented in healthy adults [32]. The SLST has demonstrated good-to-excellent reliability across diverse populations, including excellent inter-rater reliability in healthy adults under both eyes open (ICC = 0.95) and eyes closed (ICC = 0.83) conditions [32], and excellent inter-rater reliability in uninjured athletes (κ = 0.90) [45].

**Figure 3:**
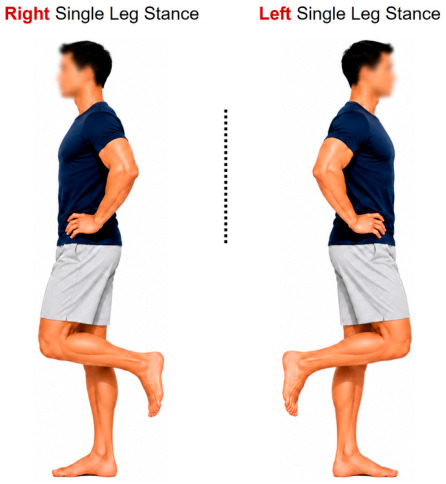
Participant positioning during single-leg stance test (SLST) assessment. Participants stood barefoot with their hands placed on the iliac crests, the stance knee maintained in a neutral extended position, and the non-stance knee flexed to approximately 90°.

Participants stood barefoot on one limb with their hands placed on the iliac crests, the stance knee maintained in a neutral extended position, and the non-stance knee flexed to approximately 90° (Figure 3). Testing was performed under both eyes open and eyes closed conditions for the right and left limbs. During eyes open trials, participants focused on a fixed visual target positioned at eye level approximately 2 m ahead of the participant. During eyes closed trials, participants initially focused on the same target before closing their eyes at the start of testing. For each trial, participants were instructed to “stand as still as possible” while maintaining the testing position.

To ensure participant safety, a chair was positioned in front of the participant, and an assessor remained nearby throughout testing. Prior to formal assessment, participants completed one familiarisation trial for each of the four testing conditions: right leg eyes open, right leg eyes closed, left leg eyes open, and left leg eyes closed. Formal assessment subsequently consisted of three trials under each of these four testing conditions, with a maximum trial duration of 30 s per trial. The mean SLST duration across the three trials for each testing condition was used for statistical analyses. A standardised 30 s rest interval was provided between consecutive trials to minimise the potential influence of fatigue.

SLST performance was quantified as the duration (seconds) participants were able to maintain unilateral stance without loss of balance or the occurrence of a compensatory movement. The test was terminated upon displacement or rotation of the stance foot, contact of the raised foot with the floor or stance limb, removal of the hands from the iliac crests, opening of the eyes during eyes closed trials, or attainment of the maximum 30 s trial duration [32,52]. Both trials completed to 30 s and trials terminated before reaching the maximum duration were retained for analysis, with the elapsed time at test termination recorded as the outcome measure. SLST duration was used as the primary outcome variable. Similar SLST protocols using stance duration as the outcome measure have previously been implemented in healthy young adults [32,34,53].

#### 2.4.5. Walking Performance

**Figure 4:**
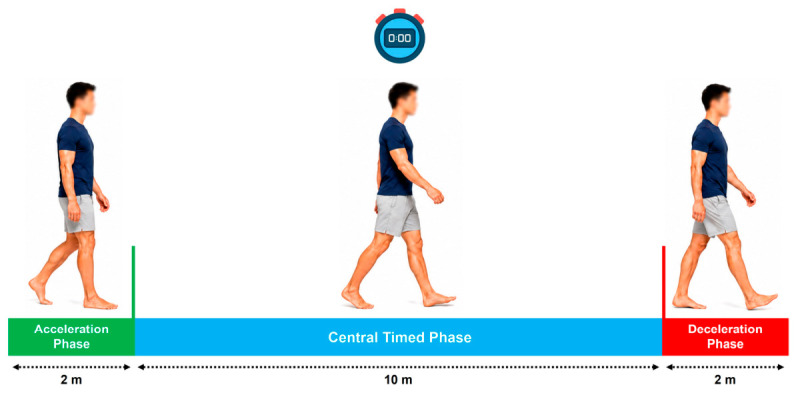
Walkway configuration for the 10 m walk test (10-MWT), comprising a 14 m pathway with a 2 m acceleration phase, a central 10 m timed section, and a 2 m deceleration phase.

Walking performance was assessed as a secondary outcome variable using the 10-MWT to quantify habitual walking speed and locomotor performance [41]. Previous studies have demonstrated excellent test–retest reliability of the 10-MWT (ICC = 0.97–0.98) in both healthy young adults [54] and older adults [41]. Participants walked at their usual comfortable pace along a standardised 14 m walkway comprising a 2 m acceleration phase, a central 10 m timed section, and a 2 m deceleration phase (Figure 4). Walking time was recorded using a stopwatch and commenced when the lead foot crossed the 2 m mark and terminated when the lead foot crossed the 12 m mark, corresponding to the beginning and end of the central 10 m timed section, respectively [41]. Both 10-MWT completion time (seconds) and walking speed (m/s) were derived from the assessment, with walking speed calculated by dividing the 10 m walking distance by the recorded completion time.

#### 2.4.6. Lower Limb Functional Capacity

**Figure 5:**
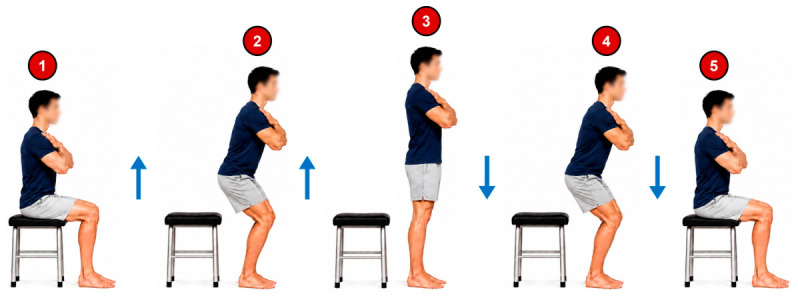
Movement sequence during the 30 s chair stand test (30 s-CST), illustrating (1) the seated starting position, (2) the sit-to-stand phase, (3) the full standing position, (4) the stand-to-sit phase, and (5) the seated ending position.

Lower limb functional capacity was assessed as a secondary outcome variable using the 30 s-CST. Previous studies have demonstrated good-to-excellent test–retest reliability of the 30 s-CST (ICC = 0.84–0.94) across diverse populations [40,55]. Participants were seated on a standardised chair with the hips and knees positioned at approximately 90° of flexion and the feet fully supported on the floor. Chair height was adjusted when necessary to maintain standardised lower limb positioning across participants. Prior to formal assessment, participants received standardised instructions and completed one practice trial. Participants were instructed to sit with their arms crossed over the chest and to perform as many sit-to-stand repetitions as possible within 30 s by repeatedly rising to a full standing position and returning to a fully seated position (Figure 5). A repetition was considered successful only when a complete sit-to-stand-to-sit cycle was achieved. The total number of completed repetitions was recorded as the outcome measure, with incomplete final repetitions counted if more than 50% of the movement cycle had been completed at the end of the test period [42].

### 2.5. Power Analysis

A post hoc power analysis was conducted using G*Power (version 3.1.9.7; Heinrich Heine University Düsseldorf, Düsseldorf, Germany) to evaluate the adequacy of the final analytical sample. Because the primary analyses examined associations between continuous exposure variables and continuous outcome measures while adjusting for continuous covariates and categorical factors, the analysis was based on an F-test within a multivariable modelling framework using the R^2^ increase approach. The model specification included one tested predictor representing the exposure variable and five adjustment variables comprising age, BMI, sex, athletic status, and limb dominance, resulting in six total predictors. Assuming a moderate effect size (f^2^ = 0.15), a significance level of 0.05, and the final analytical sample of 104 participants, the achieved statistical power was 97%. This finding supports the adequacy of the final sample size for the adjusted association analyses performed in this study.

### 2.6. Data Analysis

#### 2.6.1. Descriptive Analyses

Participant demographic, anthropometric, athletic status, and dominance characteristics were summarised for the overall sample and stratified by sex. Normally distributed continuous variables were reported as mean ± standard deviation (SD), whereas non-normally distributed continuous variables were summarised as median and interquartile range (IQR). Categorical variables were presented as frequencies and percentages. Comparisons between male and female participants were performed using independent two-sample *t*-tests for normally distributed continuous variables, Wilcoxon rank-sum tests for non-normally distributed continuous variables, and Fisher’s exact tests for categorical variables.

Descriptive statistics were generated for all primary and secondary exposure and outcome variables. Paired *t*-tests were used to compare sitting and standing navicular height measurements, right and left SSNDT scores, right and left handgrip strength values, and eyes open and eyes closed SLST durations. Sex-specific differences in the primary and secondary exposure variables were examined using independent two-sample *t*-tests.

#### 2.6.2. Generalised Estimating Equations (GEEs)

GEEs were used to examine the associations of SSNDT score and handgrip strength with SLST duration because SLST performance was assessed repeatedly under two visual conditions (eyes open and eyes closed) within the same participant. Separate GEE models were constructed for the right and left feet. In separate analyses, SLST duration was specified as the dependent variable, whereas either SSNDT score or handgrip strength was entered as the exposure variable. Visual condition was included as an independent factor in all models. To determine whether the associations between the exposure variables and SLST duration differed according to visual condition, interaction terms between the exposure variable and visual condition were initially incorporated into the models. When interaction terms were not statistically significant, they were removed from the final models and only the main effects were retained.

Models examining SSNDT score were adjusted for age [56], sex [56], BMI [56,57], athletic status [16,58], and dominant leg [59], whereas models examining handgrip strength were adjusted for age [46], sex [46], BMI [46], athletic status [46], and dominant hand [47,60]. These variables were included as potential confounders because previous evidence suggests that they may influence foot posture, muscular strength, balance performance, and physical function. An exchangeable working correlation structure was specified to account for the correlation between repeated SLST observations obtained from the same participant under the two visual conditions. GEE models were fitted using a Gaussian family with an identity link function, and robust standard errors were used for statistical inference. Results are presented as GEE coefficients with corresponding 95% confidence intervals (CIs) and *p* values. Coefficient plots were generated to visually present adjusted associations between the exposure variables and SLST duration together with the estimated effects of included covariates and factors.

#### 2.6.3. Multivariable Linear Regression

For outcomes involving a single observation per participant, multivariable linear regression models with robust standard errors were performed to examine the associations of SSNDT score and handgrip strength with 10-MWT walking speed and 30 s-CST performance. Separate models were developed for each exposure variable and outcome measure. For SSNDT models, analyses were adjusted for age [56], sex [56], BMI [56,57], athletic status [16,58], and dominant leg [59]. For handgrip strength models, analyses were adjusted for age [46], sex [46], BMI [46], athletic status [46], and dominant hand [47,59].

Both unadjusted and adjusted analyses were performed. Results are presented as regression coefficients with corresponding 95% CIs and *p* values. Model explanatory performance was quantified using the coefficient of determination (R^2^). Significant associations identified for included covariates and categorical factors were additionally reported. Detailed adjusted regression results for each exposure variable and outcome measure are presented in tabular format.

#### 2.6.4. Statistical Significance

Statistical significance was set at *p* < 0.05. Statistical analyses were performed using Stata/MP version 17 (StataCorp LLC, College Station, TX, USA). Statistical figures and graphical visualisations were generated using Stata and Microsoft Excel. Study flow diagrams were created using Microsoft PowerPoint. Illustrative figures used to visually demonstrate the exposure and outcome assessment procedures were created using an artificial intelligence-assisted image generation platform and subsequently reviewed and refined by the research team.

## 3. Results

### 3.1. Participant Selection

**Figure 6:**
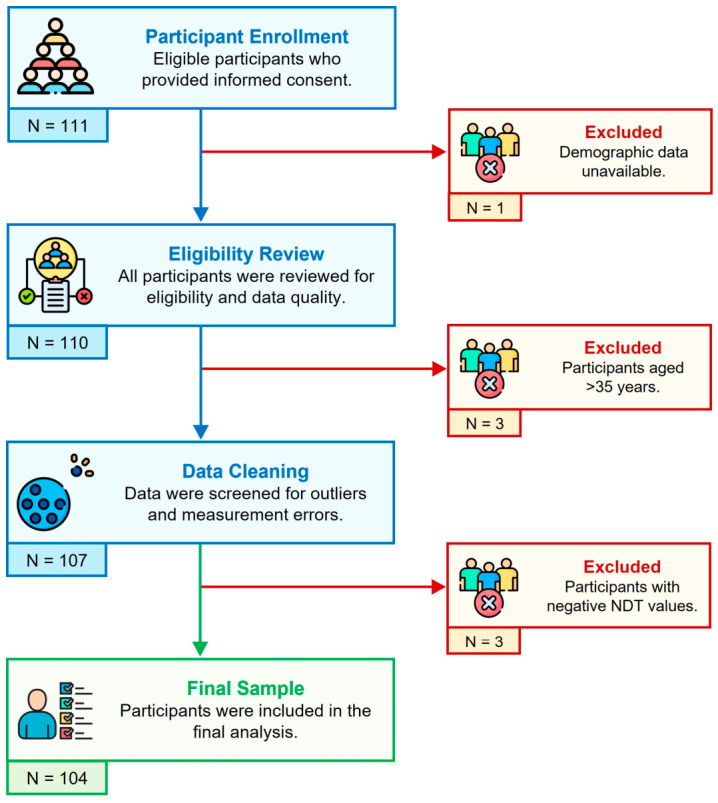
Flow diagram of participant recruitment and selection.

Overall, 111 participants fulfilled the eligibility criteria, agreed to participate, and provided informed consent. During data verification, demographic data for one participant were unavailable and were therefore excluded from the analysis. The remaining 110 participants were subsequently reviewed for eligibility and data quality. Three participants were excluded because they were older than 35 years, whereas the remaining sample consisted primarily of young adults aged between 19 and 25 years. This exclusion was applied to minimise the potential confounding influence of age on the study outcomes and to maintain greater sample homogeneity. No participants reported a history of major surgery, significant injury, or any other predefined exclusion criteria. Following these procedures, 107 participants remained eligible for inclusion. Subsequently, three participants were excluded because of implausible SSNDT scores (negative values), indicating a measurement or data-entry error. A flow diagram of participant recruitment and selection is presented in Figure 6.

### 3.2. Participant Characteristics

The final analytical sample comprised 104 participants with a mean age of 21.1 ± 1.2 years. Of the included participants, 55 (52.9%) were female and 49 (47.1%) were male. Participants had a mean standing height of 164.2 ± 9.2 cm. The median body weight was 63.3 kg (IQR: 53.3–79.9 kg), and the median BMI was 24.5 kg/m^2^ (IQR: 20.3–29.0 kg/m^2^). All participants were Saudi nationals, unmarried, residing in Makkah, and enrolled as undergraduate students at the university at the time of data collection.

Male participants demonstrated significantly greater standing height, body weight, and BMI than female participants (all *p* < 0.01), whereas age did not differ between sexes. A greater proportion of males were classified as athletic (46.9% vs. 20.0%; *p* = 0.006), while no significant sex differences were observed for dominant leg or hand dominance. Detailed participant characteristics are presented in Table 1.

### 3.3. Descriptive Statistics

#### 3.3.1. Exposure Variables

**Figure 7:**
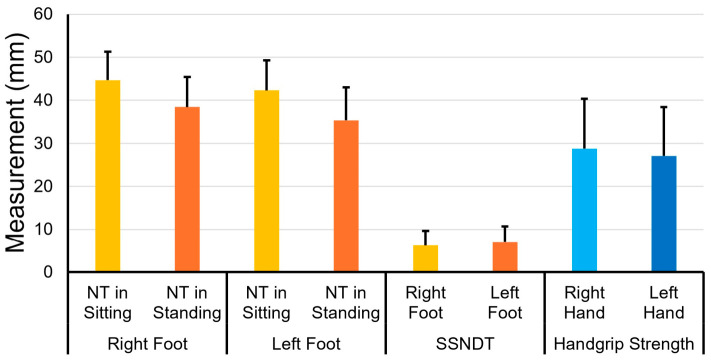
Mean navicular tuberosity (NT) height measurements in sitting and standing positions, SSNDT scores for the right and left feet, and handgrip strength measurements for the right and left hands. Error bars represent standard deviations.

The mean SSNDT score was 6.22 ± 3.42 mm for the right foot and 6.96 ± 3.76 mm for the left foot. A statistically significant side-to-side difference was identified, with higher SSNDT scores observed for the left foot compared with the right foot (*p* = 0.043). However, the magnitude of the difference was small, with a mean between-foot difference of less than 1 mm (Figure 7, Table 2). Handgrip strength demonstrated mean values of 28.76 ± 11.59 kg for the right hand and 27.07 ± 11.39 kg for the left hand, with significantly greater handgrip strength (*p* = 0.001; Figure 7, Table 2) observed on the right side compared with the left side. Detailed descriptive statistics for the exposure variables are presented in Table 2.

Sex-specific analyses demonstrated significant differences in the exposure variables. For SSNDT, males demonstrated significantly higher scores than females for the right foot (7.25 ± 3.71 mm vs. 5.33 ± 2.89 mm, *p* = 0.004). Although males also exhibited higher SSNDT scores than females for the left foot (7.50 ± 3.64 mm vs. 6.49 ± 3.83 mm), this difference did not reach statistical significance. For handgrip strength, males exhibited significantly greater strength than females for both the right hand (38.60 ± 9.01 kg vs. 20.00 ± 4.23 kg, *p* < 0.001) and the left hand (36.22 ± 9.96 kg vs. 18.93 ± 3.98 kg, *p* < 0.001).

#### 3.3.2. Outcome Variables

**Figure 8:**
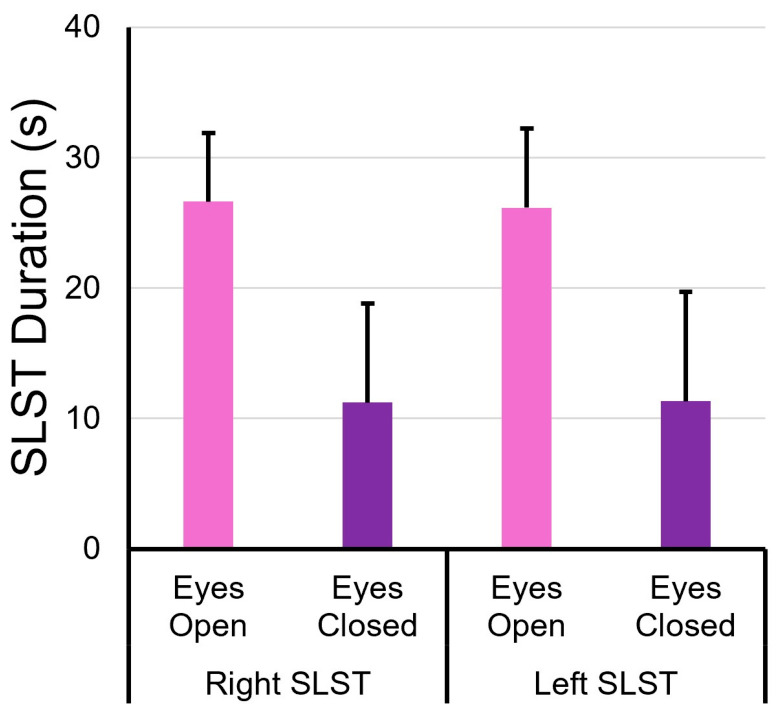
Mean single-leg stance test (SLST) duration under eyes open and eyes closed conditions for the right and left limbs. Error bars represent standard deviations.

For the primary outcome variable, the mean SLST durations for the right side under the eyes open and eyes closed conditions were 26.62 ± 5.28 s and 11.22 ± 7.58 s, respectively. For the left side, the corresponding values were 26.16 ± 6.09 s and 11.32 ± 8.39 s, respectively. The differences in SLST duration between the eyes open and eyes closed conditions were statistically significant (both *p* < 0.001) for both the right and left sides (Figure 8, Table 2). For the secondary outcome variables, the mean completion time for the 10-MWT was 8.61 ± 1.07 s, corresponding to a mean walking speed of 1.18 ± 0.15 m/s, while the mean 30 s-CST performance was 10.99 ± 2.14 repetitions. Detailed descriptive statistics for the outcome measures are presented in Table 2.

### 3.4. Associations Between Foot Arch Status and Outcome Variables

#### 3.4.1. Association Between SSNDT Score and SLST Performance

**Figure 9:**
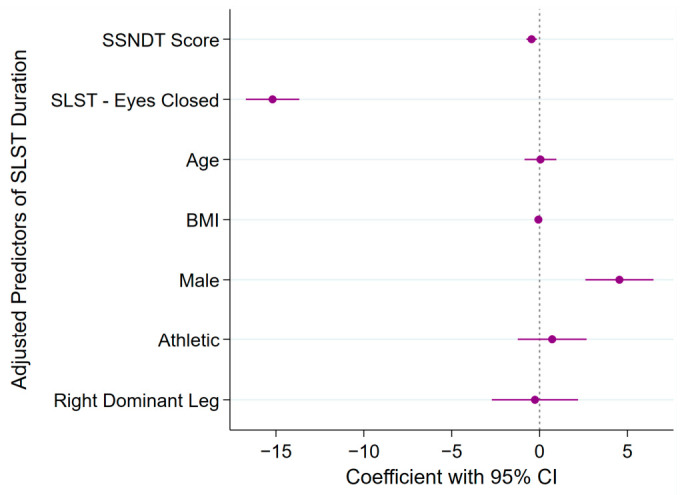
Coefficient plot showing adjusted coefficients (dots) and 95% confidence intervals (CIs; horizontal lines) from the GEE model examining the association between right sit-to-stand navicular drop test (SSNDT) score and single-leg stance test (SLST) duration for the right foot after adjustment for age, sex, body mass index (BMI), athletic status, and dominant leg. The vertical dashed line represents the null effect (β = 0).

**Figure 10:**
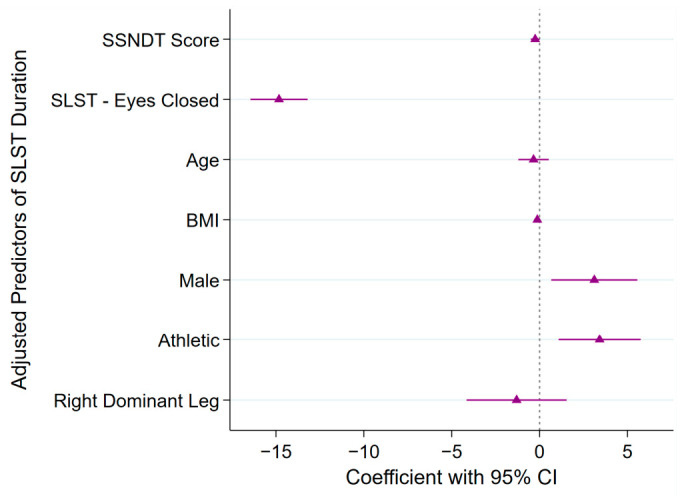
Coefficient plot showing adjusted coefficients (triangles) and 95% confidence intervals (CIs; horizontal lines) from the GEE model examining the association between left sit-to-stand navicular drop test (SSNDT) score and single-leg stance test (SLST) duration for the left foot after adjustment for age, sex, body mass index (BMI), athletic status, and dominant leg. The vertical dashed line represents the null effect (β = 0).

The adjusted GEE models demonstrated significant overall model fit, with an overall Wald χ^2^ statistic of 695.74 (*p* < 0.001) for the model examining the association between right SSNDT score and right SLST duration and 634.40 (*p* < 0.001) for the corresponding model examining the association between left SSNDT score and left SLST duration. The interaction term between SSNDT score and visual condition was not statistically significant for either foot and was therefore excluded from the final models. In the adjusted GEE models, greater SSNDT scores were independently associated with poorer SLST performance for both feet. For the right foot, each 1 mm increase in SSNDT score was associated with a 0.46 s reduction in SLST duration (β = −0.46, 95% CI: −0.75 to −0.16, *p* = 0.003; Figure 9). Similarly, each 1 mm increase in SSNDT score for the left foot was associated with a 0.25 s reduction in SLST duration (β = −0.25, 95% CI: −0.50 to −0.00, *p* = 0.048; Figure 10). Visual condition demonstrated a substantial independent association with SLST performance in both models. Compared with the eyes open condition, the eyes closed condition was associated with a significantly shorter SLST duration for the right foot (β = −15.19, 95% CI: −16.72 to −13.67, *p* < 0.001; Figure 9) and the left foot (β = −14.83, 95% CI: −16.45 to −13.20, *p* < 0.001; Figure 10).

Among the included covariates and factors, sex was significantly associated with SLST performance in both models, with male participants demonstrating longer SLST duration than female participants for the right foot (β = 4.54, 95% CI: 2.61 to 6.48, *p* < 0.001; Figure 9) and the left foot (β = 3.11, 95% CI: 0.66 to 5.56, *p* = 0.013; Figure 10). Athletic status was additionally significantly associated with left foot SLST performance, with athletic participants demonstrating longer SLST duration than non-athletic participants (β = 3.42, 95% CI: 1.08 to 5.75, *p* = 0.004; Figure 10). No significant associations were observed for age, BMI, or leg dominance in either GEE model.

#### 3.4.2. Association of SSNDT Score with 10-MWT and 30 s-CST Performance

No significant associations were observed between SSNDT score and 10-MWT walking speed for either the right or left foot in either the unadjusted or adjusted regression analyses (all *p* > 0.05). Similarly, no significant associations (Figures S1 and S2) were identified for age, BMI, sex, athletic status, or leg dominance in any of the adjusted models (all *p* > 0.05). The adjusted regression models including right and left SSNDT scores explained 6.8% (R^2^ = 0.0678) and 6.4% (R^2^ = 0.0643) of the variance in 10-MWT walking speed, respectively.

**Figure 11:**
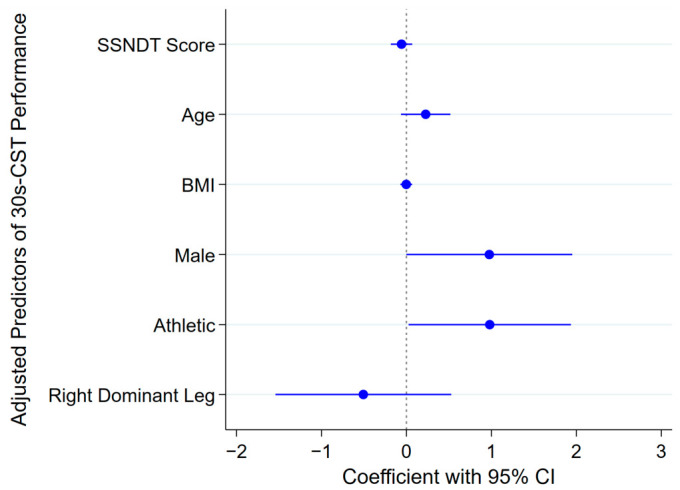
Coefficient plot showing adjusted coefficients (dots) and 95% confidence intervals (CIs; horizontal lines) from the multivariable linear regression model examining the association between right sit-to-stand navicular drop test (SSNDT) score and 30 s chair stand test (30 s-CST) performance after adjustment for age, sex, body mass index (BMI), athletic status, and dominant leg. The vertical dashed line represents the null effect (β = 0).

**Figure 12:**
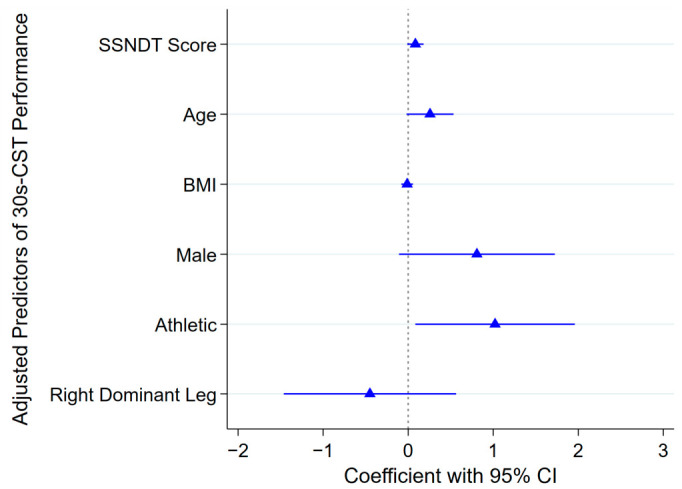
Coefficient plot showing adjusted coefficients (triangles) and 95% confidence intervals (CIs; horizontal lines) from the multivariable linear regression model examining the association between left sit-to-stand navicular drop test (SSNDT) score and 30 s chair stand test (30 s-CST) after adjustment for age, sex, body mass index (BMI), athletic status, and dominant leg. The vertical dashed line represents the null effect (β = 0).

No significant associations were observed between SSNDT score and 30 s-CST performance for either the right or left foot in the unadjusted regression analyses (all *p* > 0.05). Similarly, in the adjusted regression analyses (Figure 11 and Figure 12), SSNDT score was not significantly associated with 30 s-CST performance for either the right foot (*p* = 0.366) or the left foot (*p* = 0.081). Athletic status was the only factor significantly associated with 30 s-CST performance in both adjusted models, with athletic participants demonstrating a greater number of chair-stand repetitions than non-athletic participants for the right foot (β = 0.98, 95% CI: 0.03 to 1.94, *p* = 0.044; Figure 11) and the left foot (β = 1.02, 95% CI: 0.08 to 1.96, *p* = 0.033; Figure 12). The adjusted regression models including right and left SSNDT scores explained 15.6% (R^2^ = 0.1564) and 17.1% (R^2^ = 0.1714) of the variance, respectively.

### 3.5. Associations Between Handgrip Strength and Outcome Variables

#### 3.5.1. Association Between Handgrip Strength and SLST Performance

**Figure 13:**
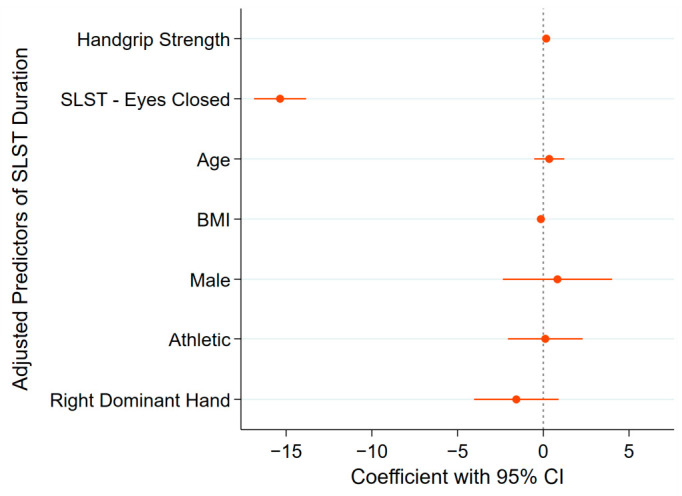
Coefficient plot showing adjusted coefficients (dots) and 95% confidence intervals (CIs; horizontal lines) from the GEE model examining the association between right handgrip strength and SLST duration for the right foot after adjustment for age, sex, body mass index (BMI), athletic status, and dominant hand. The vertical dashed line represents the null effect (β = 0).

**Figure 14:**
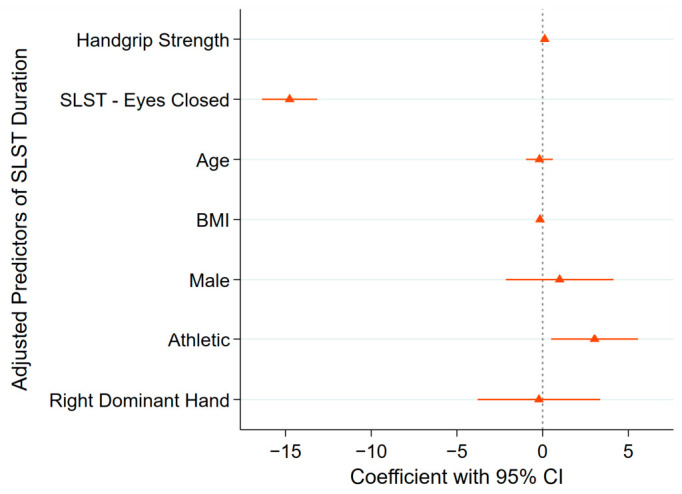
Coefficient plot showing adjusted coefficients (triangles) and 95% confidence intervals (CIs; horizontal lines) from the GEE model examining the association between left handgrip strength and SLST duration for the left foot after adjustment for age, sex, body mass index (BMI), athletic status, and dominant hand. The vertical dashed line represents the null effect (β = 0).

The adjusted GEE models demonstrated significant overall model fit, with an overall Wald χ^2^ statistic of 565.36 (*p* < 0.001) for the model examining the association between right handgrip strength and right SLST duration and 537.56 (*p* < 0.001) for the corresponding model examining the association between left handgrip strength and left SLST duration. The interaction between handgrip strength and visual condition was not statistically significant for either hand and was therefore excluded from the final models. In the adjusted GEE models, greater handgrip strength was independently associated with better SLST performance for the right foot but not the left foot. For the right foot, each 1 kg increase in right handgrip strength was associated with a 0.17 s increase in SLST duration (β = 0.17, 95% CI: 0.03 to 0.31, *p* = 0.019; Figure 13). Visual condition demonstrated a substantial association with SLST duration in both models. Compared with the eyes open condition, the eyes closed condition was associated with significantly shorter SLST duration for the right foot (β = −15.35, 95% CI: −16.87 to −13.83, *p* < 0.001; Figure 13) and the left foot (β = −14.76, 95% CI: −16.37 to −13.14, *p* < 0.001; Figure 14).

Among the included covariates and factors, athletic status was significantly associated with SLST duration for the left foot, with athletic participants demonstrating longer SLST duration than non-athletic participants (β = 3.03, 95% CI: 0.50 to 5.56, *p* = 0.019; Figure 14). No significant associations were observed for age, BMI, sex, or hand dominance in either GEE model.

#### 3.5.2. Association of Handgrip Strength with 10-MWT and 30 s-CST Performance

For 10-MWT walking speed, greater handgrip strength was associated with faster walking speed for both the right (β = 0.0026, 95% CI: 0.0003 to 0.0050, *p* = 0.026) and left (β = 0.0027, 95% CI: 0.0003 to 0.0051, *p* = 0.025) feet in the unadjusted analyses. After adjustment, the associations between handgrip strength and walking speed were no longer statistically significant (Figures S3 and S4). No covariates or factors were significantly associated with walking speed (all *p* > 0.05). The adjusted regression models including right and left handgrip strength explained 11.3% (R^2^ = 0.1127) and 10.3% (R^2^ = 0.1028) of the variance in 10-MWT walking speed, respectively.

**Figure 15:**
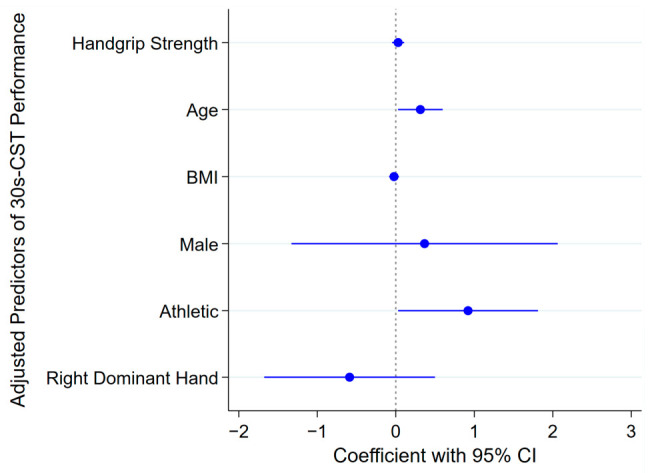
Coefficient plot showing adjusted coefficients (dots) and 95% confidence intervals (CIs; horizontal lines) from the multivariable linear regression model examining the association between right handgrip strength and 30 s chair stand test (30 s-CST) performance after adjustment for age, sex, body mass index (BMI), athletic status, and dominant leg. The vertical dashed line represents the null effect (β = 0).

**Figure 16:**
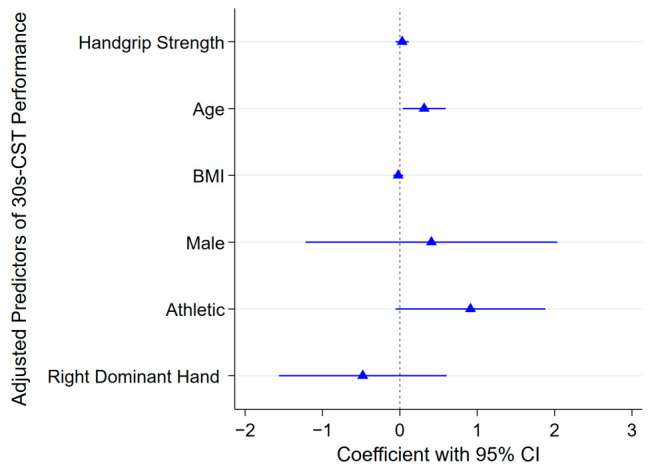
Coefficient plot showing adjusted coefficients (triangles) and 95% confidence intervals (CIs; horizontal lines) from the multivariable linear regression model examining the association between left handgrip strength and 30 s chair stand test (30 s-CST) after adjustment for age, sex, body mass index (BMI), athletic status, and dominant leg. The vertical dashed line represents the null effect (β = 0).

For 30 s-CST performance, greater handgrip strength was associated with better 30 s-CST performance for both the right (β = 0.05, 95% CI: 0.01 to 0.09, *p* = 0.016) and left (β = 0.05, 95% CI: 0.01 to 0.10, *p* = 0.028) feet in the unadjusted analyses. However, after adjustment, the associations between handgrip strength and 30 s-CST performance were no longer statistically significant. Among the covariates and factors included in the adjusted models, age was significantly associated with 30 s-CST performance in both models. Specifically, each one-year increase in age was associated with a 0.31-repetition increase in 30 s-CST performance for the right foot (β = 0.31, 95% CI: 0.03 to 0.60, *p* = 0.031; Figure 15) and the left foot (β = 0.31, 95% CI: 0.04 to 0.59, *p* = 0.027; Figure 16). Athletic status was also significantly associated with 30 s-CST performance in the right-side model, with athletic participants performing more chair-stand repetitions than non-athletic participants (β = 0.92, 95% CI: 0.03 to 1.81, *p* = 0.043; Figure 15). No significant associations were observed for BMI, sex, or hand dominance in either adjusted model. The adjusted regression models including right and left handgrip strength explained 16.3% (R^2^ = 0.1625) and 16.4% (R^2^ = 0.1637) of the variance in 30 s-CST performance, respectively.

## 4. Discussion

### 4.1. Main Findings

This study investigated the associations of foot arch status and handgrip strength with unilateral balance performance, walking performance, and lower limb functional capacity in healthy young Saudi adults. The principal finding was that greater SSNDT scores were independently associated with poorer unilateral balance performance for both feet, indicating that greater functional lowering of the MLA during weight bearing was associated with reduced balance capacity. In contrast, handgrip strength demonstrated a more limited association with unilateral balance performance, with a significant association observed only between right handgrip strength and right-foot balance performance. Neither SSNDT score nor handgrip strength was independently associated with 10-MWT outcomes or 30 s-CST performance after adjustment for potential confounding factors. Unilateral balance performance was also markedly reduced under eyes closed conditions, highlighting the important contribution of visual input to postural balance control. Additionally, athletic status demonstrated the most consistent associations with balance and physical performance outcomes, whereas sex and age were associated with only a limited number of outcomes.

### 4.2. Statistical Considerations

The explanatory ability of the multiple linear regression models should also be considered when interpreting the findings. Although the adjusted regression models for 30 s-CST explained approximately 16–17% of the variance in performance, the adjusted models for 10-MWT walking speed explained less than 10% of the outcome variance, indicating more limited explanatory ability. Therefore, these findings should be interpreted with appropriate caution. The modest explanatory ability of these models also suggests that additional biomechanical, neuromuscular, sensory, psychological, and behavioural factors not evaluated in the present study may contribute to balance and physical performance outcomes. For the GEE models, the coefficients should be interpreted in the context of the measurement scale of the exposure variables, particularly SSNDT, which was measured in millimetres. Consequently, each coefficient represents the expected change in SLST duration associated with a 1 mm increase in navicular drop, resulting in numerically modest coefficient estimates despite statistically significant associations. Accordingly, larger differences in SSNDT scores would be expected to correspond to proportionally larger estimated differences in SLST duration. These estimates quantify statistical associations and should not be interpreted as established thresholds of clinical significance.

### 4.3. Comparison of Descriptive Findings with Previous Studies

Mean SSNDT scores were 6.22 ± 3.42 mm for the right foot and 6.96 ± 3.76 mm for the left foot, both within the commonly accepted normal navicular drop range of approximately 5–9 mm [6,8,15]. Although a statistically significant side-to-side difference was observed, the mean difference was less than 1 mm and is unlikely to be clinically meaningful, possibly reflecting normal biological variation between limbs. The observed SSNDT values were broadly comparable to those reported in healthy adults from Spain [6], Slovenia [61], Poland [62], and the United States [63], although some variation between studies is expected because of differences in participant characteristics and measurement procedures.

Handgrip strength averaged 28.76 ± 11.59 kg for the right hand and 27.07 ± 11.39 kg for the left hand, with significantly greater values observed in the right hand and among males. This pattern is consistent with previous Saudi normative studies reporting higher handgrip strength in males and peak strength during young adulthood [64,65]. Although the observed values were modestly lower than some age-matched Saudi reference data [64], they were broadly comparable to those reported in healthy young adults, supporting the representativeness of the study sample.

### 4.4. Associations of SSNDT and Handgrip Strength with Balance Performance

Greater SSNDT scores were independently associated with poorer unilateral balance performance, supporting the hypothesis that MLA behaviour plays a role in postural balance control. In contrast, handgrip strength demonstrated a more limited association, with a significant relationship observed only for the right foot after adjustment. These findings suggest that foot arch mobility may exert a stronger and more consistent influence on unilateral balance performance than overall muscular strength in healthy young adults. They are also consistent with previous evidence linking pronated foot posture to impaired postural balance control, including greater postural sway during bilateral [66] and unilateral standing [66,67], greater centre of pressure displacement during the transition from double-leg to single-leg stance [68], and reduced dynamic stability characterised by greater postural corrections and longer time to stabilisation following landing tasks [69].

The observed associations may also reflect the greater postural demands of unilateral balance tasks. Compared with bilateral standing, single-leg stance requires greater neuromuscular control to maintain the body’s centre of mass over a reduced base of support [36,37,70] and is therefore more sensitive to subtle impairments in postural balance and musculoskeletal function [34]. Consequently, alterations in foot function that have little influence during routine activities may become more apparent during unilateral balance tasks, which may explain why SSNDT was associated with SLST but not with walking speed or chair-stand performance.

In addition to foot arch behaviour, muscular strength may also contribute to postural balance performance. The present study demonstrated a significant association between handgrip strength and SLST performance. Previous evidence suggests that impaired unilateral balance reflects reduced muscular strength and neuromuscular control capacity [33], with positive associations reported between single-leg balance performance and lower limb muscle strength, including the hip flexors, hip extensors, hip abductors, knee extensors, and ankle plantar flexors [36,70,71]. As handgrip strength has been proposed as a practical surrogate of overall muscular strength, physical function, and general health status [27,29,72,73], the observed association is biologically plausible. Previous studies have also reported associations between handgrip strength and quadriceps strength in young adults [30], hip, knee, and ankle muscle strength in community-dwelling older adults [31], and postural control in community-dwelling older adults [31]. These findings suggest that greater overall muscular capacity may reflect superior postural stabilisation during challenging unilateral balance tasks. However, because this association remained significant only for the right side after adjustment, muscular strength appears to contribute less consistently to unilateral balance performance than foot arch behaviour in the present cohort. The observed right-sided association should be interpreted with caution, as the predominance of right-hand and right-leg dominance in the study population may have contributed to the observed asymmetry through greater functional use of the dominant limbs, potentially influencing both muscular performance and postural control.

### 4.5. Associations of SSNDT and Handgrip Strength with Physical Performance

#### 4.5.1. 10-MWT

In contrast to the findings for SLST performance, neither foot arch status nor handgrip strength was independently associated with 10-MWT walking speed after adjustment for potential confounders. Although greater handgrip strength was associated with faster walking performance in the unadjusted analyses, this association was attenuated after adjustment, whereas SSNDT score was not associated with walking speed in either the unadjusted or adjusted models. These findings suggest that habitual walking speed in healthy young adults may be relatively insensitive to subtle variations in foot arch behaviour and general muscular strength. Furthermore, because habitual walking speed is generally well preserved in healthy young adults, the limited variability in this outcome may have reduced the ability to detect independent associations. This interpretation is consistent with previous evidence showing that SSNDT is unrelated to walking velocity and step length [14] and that the effects of foot posture are more evident in gait kinematics, kinetics, and plantar pressure characteristics than in walking speed itself [10,74,75]. Although greater handgrip strength has been associated with faster walking speed in older adults [76,77,78] and clinical populations [79,80], these findings may not be generalisable to healthy young adults because of differences in the demographic and clinical characteristics.

#### 4.5.2. 30 s-CST

Neither foot arch status nor handgrip strength demonstrated independent associations with 30 s-CST performance after adjustment for potential confounders. Although greater handgrip strength was associated with better chair-stand performance in the unadjusted analyses, this association was no longer significant following adjustment, suggesting that neither MLA behaviour nor general muscular strength independently explained repeated sit-to-stand performance in this young cohort. The absence of associations with either SSNDT score or handgrip strength may reflect the distinct demands of the 30 s-CST, which is a bilateral functional task primarily dependent on lower limb strength, muscular endurance, movement coordination, and repeated force generation [40,42], rather than the postural control demands characteristic of the SLST. Consequently, subtle variations in MLA behaviour may have limited influence on chair-stand performance, whereas direct measures of lower limb strength and muscular endurance may provide more relevant measures of physical capacity than handgrip strength. Together with the 10-MWT findings, these results suggest that the functional contributions of foot arch behaviour and general muscular strength may be more apparent during tasks requiring substantial unilateral postural balance control than during routine bilateral functional activities in healthy young adults. Consequently, unilateral balance assessment may be better suited to detecting these subtle functional differences in healthy young adults.

### 4.6. Effects of Covariates and Factors

The marked reduction in SLST duration under eyes closed conditions highlights the importance of visual input for unilateral balance. Across both the SSNDT and handgrip strength models, removal of visual input was associated with substantially poorer balance performance, consistent with previous studies demonstrating marked deterioration in single-leg balance when visual feedback is unavailable [32,51,81]. This finding also aligns with evidence that individuals with balance impairments, altered lower limb function, or musculoskeletal conditions rely more heavily on visual input to compensate for deficits in proprioceptive and neuromuscular control [82,83].

Athletic status emerged as the most consistent participant characteristic associated with balance and physical performance. Athletic participants demonstrated superior SLST performance in some models and completed more chair-stand repetitions than non-athletic participants. These findings are biologically plausible given the neuromuscular demands of regular sport participation and are consistent with previous research reporting sport-related differences in postural sway characteristics and single-leg balance performance [84].

Male participants demonstrated longer SLST duration than females in the SSNDT models; however, because this association was not consistently observed across all balance models, it should be interpreted cautiously. Age was positively associated with 30 s-CST performance in the handgrip models. Although the narrow age range limits interpretation, this finding is consistent with evidence that muscular performance and handgrip strength continue to increase during early adulthood [85].

Although BMI was not independently associated with any study outcome, body mass may still influence foot loading, MLA behaviour, and postural balance. Future studies involving broader BMI distributions or stratified analyses may help clarify whether these associations differ across BMI categories.

### 4.7. Clinical Implications

The present findings have several potential clinical implications. First, the consistent association between greater SSNDT scores and poorer unilateral balance performance suggests that arch mobility may be an important correlate of postural stability in healthy young adults. Consequently, assessment of arch mobility may provide clinically relevant information beyond its traditional role in evaluating lower limb biomechanics. Second, these findings suggest that the functional consequences of arch mobility extend beyond the foot, highlighting its potential contribution to postural control and lower limb function. Finally, given the widespread use of balance assessment in injury prevention, rehabilitation, and return to sport, SSNDT assessment may help identify individuals with greater arch mobility who demonstrate poorer balance performance during challenging unilateral tasks. Future longitudinal studies are warranted to determine whether the observed associations between MLA behaviour and unilateral balance performance are associated with subsequent functional outcomes or injury risk across different populations. These findings should be interpreted within the context of the study population, and their applicability to other populations requires further investigation.

### 4.8. Limitations

Several limitations should be acknowledged. First, the cross-sectional design precludes causal inference; therefore, it cannot be determined whether the observed associations between foot arch mobility, handgrip strength, and physical performance outcomes are causal or whether they are influenced by underlying biomechanical, neuromuscular, or other unmeasured factors. Although the analyses adjusted for several important covariates, residual confounding from unmeasured factors, including lower limb flexibility and joint hypermobility, cannot be excluded. Longitudinal studies are needed to clarify the temporal direction of these associations. Second, participants consisted primarily of healthy young Saudi adults recruited from a single university, which may have reduced variability in the study outcomes and limits the generalisability of the findings to older adults, clinical populations (e.g., individuals with musculoskeletal disorders), non-student populations, highly trained athletes, and populations from different cultural or geographical settings. Third, muscular strength was assessed using handgrip strength, a surrogate measure of overall muscular strength that does not directly assess lower limb muscle function. Consequently, the observed associations may underestimate the contribution of local neuromuscular factors. Future studies incorporating direct measures of lower limb strength may provide a more comprehensive understanding of their role in balance and physical performance. Fourth, although the SLST is a valid and clinically practical measure of unilateral balance, it does not capture the biomechanical and sensorimotor mechanisms underlying postural control. Instrumented assessments, such as centre of pressure or kinematic analyses, may provide additional insight into relationships not detected by clinical balance tests alone. Fifth, the maximum 30 s duration of the SLST may have introduced a ceiling effect under the eyes open condition in some healthy young participants, potentially reducing the ability of the test to discriminate higher levels of balance performance. Sixth, athletic status was included as a dichotomous variable rather than being assessed using a validated quantitative measure of habitual physical activity; therefore, the influence of activity frequency, intensity, duration, and type could not be evaluated. Seventh, although an a priori sample size calculation was not performed, the adequacy of the final analytical sample was confirmed by post hoc power analysis. Finally, although each statistical model addressed a distinct exposure–outcome association, the possibility of Type I error arising from the number of statistical tests performed cannot be completely excluded.

## 5. Conclusions

Greater SSNDT scores were independently associated with poorer unilateral balance performance in healthy young adults, indicating that greater lowering of the MLA during weight bearing is associated with reduced balance capacity. Handgrip strength demonstrated only a limited association with unilateral balance performance, with a significant association observed only for the right side. Neither SSNDT score nor handgrip strength was independently associated with walking performance or lower limb functional capacity after adjustment for confounders. Visual condition exerted a substantial influence on balance performance, whereas athletic status was consistently associated with superior balance and physical performance. Collectively, these findings suggest that functional MLA behaviour is more strongly related to unilateral balance performance than to walking speed or chair-stand performance in healthy young adults. Future studies should examine whether these associations are evident in populations with different clinical characteristics, including individuals with musculoskeletal disorders.

## Figures and Tables

**Table 1:** Participant characteristics stratified by sex.

Characteristics		Mean ± SD, Median (IQR) or N (%)			*p* Value
		Both	Male	Female	
Age		21.07 ± 1.23	21 (20–22)	21 (20–22)	0.5224
Height (cm)		164.19 ± 9.23	170.79 ± 7.75	158.31 ± 5.85	0.0000
Weight (kg)		70.50 ± 23.77	73.4 (63.3–99.9)	57.3 (48.1–65.4)	0.0000
BMI (kg/m^2^)		25.85 ± 7.35	26.2 (21.7–33.3)	22.8 (19.0–26.2)	0.0072
Athletic status	Yes	34 (32.69)	23 (46.94)	11 (20.00)	0.006
	No	70 (67.31)	26 (53.06)	44 (80.00)	
Dominant leg	Right	84 (80.77)	38 (77.55)	46 (83.64)	0.464
	Left	20 (19.23)	11 (22.45)	9 (16.36)	
Dominant hand	Right	94 (87.85)	42 (85.71)	52 (89.66)	0.566
	Left	13 (12.15)	7 (14.29)	6 (10.34)	

Abbreviations: SD, standard deviation; IQR, interquartile range; N, number of participants; %, percent; cm, centimetres; kg, kilograms; BMI, body mass index; kg/m^2^, kilograms per square metre.

**Table 2:** Descriptive statistics of exposure and outcome variables.

Variables			Mean ± SD	*p* Value
Exposure	Primary	Right NT height in sitting (mm)	44.68 ± 6.63	0.0000
		Right NT height in standing (mm)	38.46 ± 6.98	
		Left NT height in sitting (mm)	42.31 ± 6.99	0.0000
		Left NT height in standing (mm)	35.35 ± 7.68	
		Right SSNDT score (mm)	6.22 ± 3.42	0.0428
		Left SSNDT score (mm)	6.96 ± 3.76	
	Secondary	Right handgrip strength (kg)	28.76 ± 11.59	0.0009
		Left handgrip strength (kg)	27.07 ± 11.39	
Outcome	Primary	Right SLST-EO (s)	26.62 ± 5.28	0.0000
		Right SLST-EC (s)	11.22 ± 7.58	
		Left SLST-EO (s)	26.16 ± 6.09	0.0000
		Left SLST-EC (s)	11.32 ± 8.39	
	Secondary	10-MWT completion time (s)	8.61 ± 1.07	-
		10-MWT walking speed (m/s)	1.18 ± 0.15	-
		30 s-CST (reps)	10.99 ± 2.14	-

Abbreviations: SD, standard deviation; NT, navicular tuberosity; mm, millimetres; SSNDT, sit-to-stand navicular drop test; kg, kilograms; SLST, single-leg stance test; EO, eyes open; s, seconds, EC, eyes closed; 10-MWT, 10 m walk test; m/s, metres per second; 30 s-CST, 30 s chair stand test; reps, repetitions.

## Data Availability

The original contributions presented in this study are included in the article; further inquiries can be directed to the corresponding author.

## Supplementary Materials

The following supporting information can be downloaded at https://www.mdpi.com/article/10.3390/diagnostics16142279/s1, Figure S1: Adjusted association between right SSNDT and walking speed; Figure S2: Adjusted association between left SSNDT and walking speed; Figure S3: Adjusted association between right handgrip strength and walking speed; Figure S4: Adjusted association between left handgrip strength and walking speed.

## Abbreviations

The following abbreviations are used in this manuscript:

MLA	Medial longitudinal arch
SSNDT	Sit-to-stand navicular drop test
NDT	Navicular drop test
SLST	Single-leg stance test
10-MWT	10 m walk test
30 s-CST	30 s chair stand test
BMI	Body mass index
STROBE	Strengthening the Reporting of Observational Studies in Epidemiology guidelines
ICC	Intraclass correlation coefficient
SD	Standard deviation
IQR	Interquartile range
GEEs	Generalised estimating equations
CIs	Confidence intervals
R^2^	Coefficient of determination
N	Number of participants
%	Percent
cm	Centimetres
kg	Kilograms
kg/m^2^	Kilograms per square metre
NT	Navicular tuberosity
mm	Millimetres
EO	Eyes open
s	Seconds
EC	Eyes closed
m/s	Metres per second
reps	Repetitions
