# Supplementary material for: Foot Arch Status and Muscular Strength: Associations with Unilateral Balance and Physical Performance
Source: Diagnostics (Basel). 2026 Jul 21;16(14):2279. doi: 10.3390/diagnostics16142279 (PMC13409235; doi:10.3390/diagnostics16142279)
Supplement: Supplementary file 1 [file diagnostics-16-02279-s001.zip › diagnostics-4398259-supplementary.pdf]

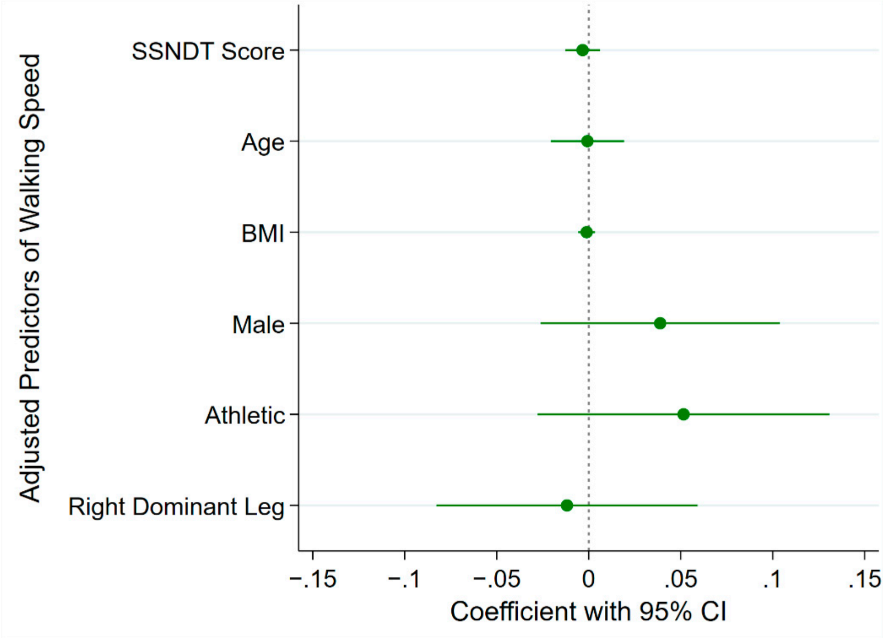

Figure S1. Coefficient plot showing adjusted coefficients (dots) and 95% confidence intervals (CIs; horizontal lines) from the multivariable linear regression model examining the association between right sit-to-stand navicular drop test (SSNDT) score and walking speed after adjustment for age, sex, body mass index (BMI), athletic status, and dominant leg. The vertical dashed line represents the null effect ($\beta = 0$).

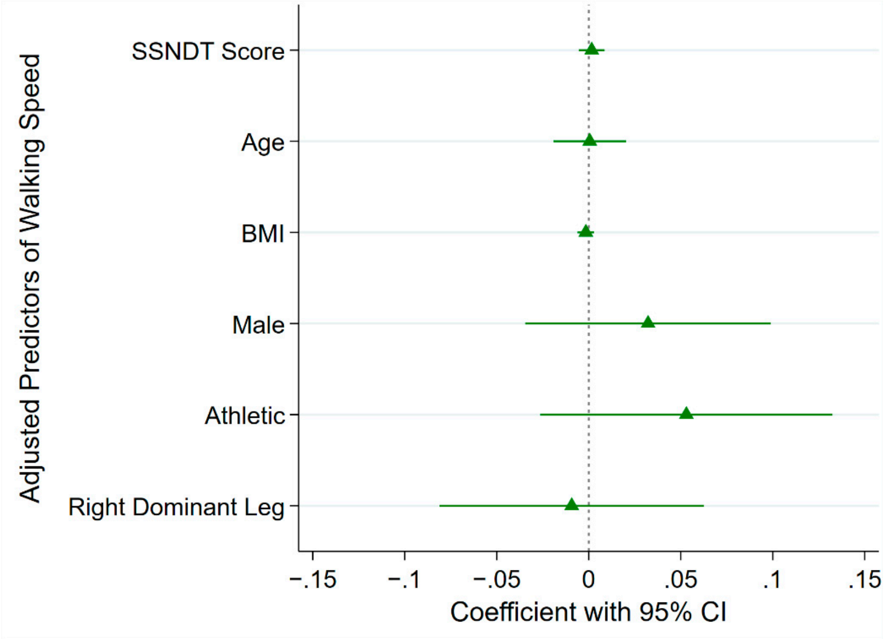

Figure S2. Coefficient plot showing adjusted coefficients (triangles) and 95% confidence intervals (CIs; horizontal lines) from the multivariable linear regression model examining the association between left sit-to-stand navicular drop test (SSNDT) score and walking speed after adjustment for age, sex, body mass index (BMI), athletic status, and dominant leg. The vertical dashed line represents the null effect ($\beta = 0$).

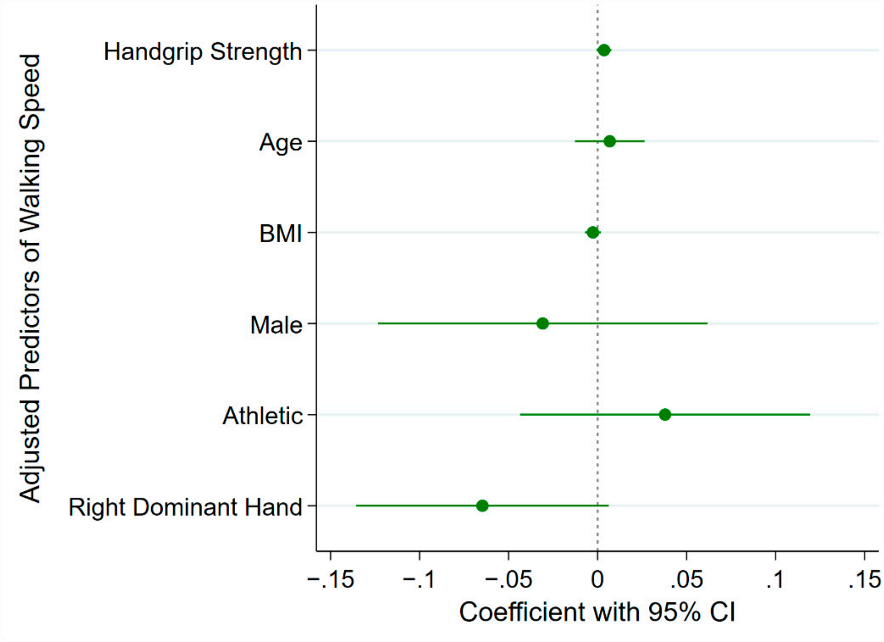

Figure S3. Coefficient plot showing adjusted coefficients (dots) and 95% confidence intervals (CIs; horizontal lines) from the multivariable linear regression model examining the association between right handgrip strength and walking speed after adjustment for age, sex, body mass index (BMI), athletic status, and dominant hand. The vertical dashed line represents the null effect ($\beta = 0$).

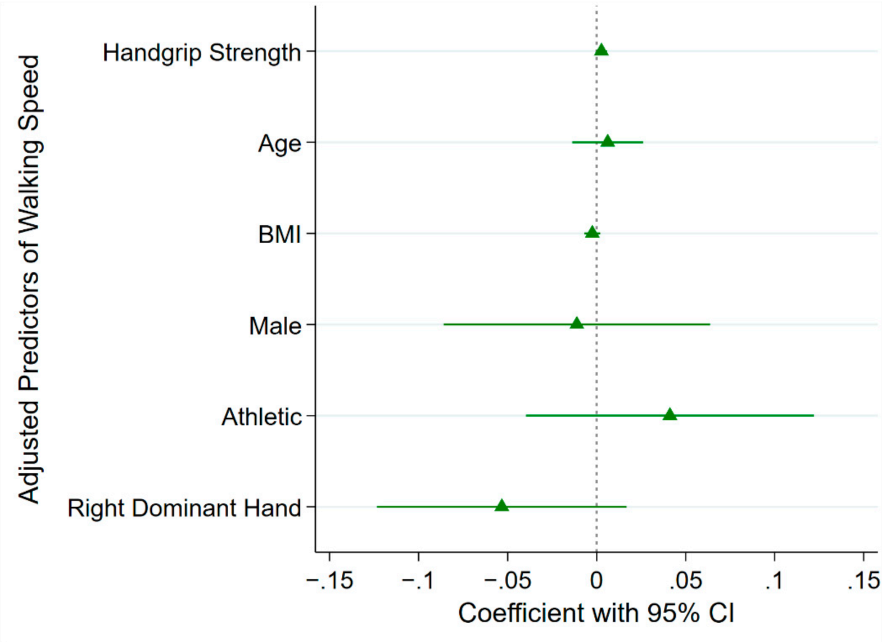

Figure S4. Coefficient plot showing adjusted coefficients (triangles) and 95% confidence intervals (CIs; horizontal lines) from the multivariable linear regression model examining the association between left handgrip strength and walking speed after adjustment for age, sex, body mass index (BMI), athletic status, and dominant hand. The vertical dashed line represents the null effect ($\beta = 0$).
